# Development of the Top Tips Habit-Based Weight Loss App and Preliminary Indications of Its Usage, Effectiveness, and Acceptability: Mixed-Methods Pilot Study

**DOI:** 10.2196/12326

**Published:** 2019-05-10

**Authors:** Nathalie Kliemann, Helen Croker, Fiona Johnson, Rebecca J Beeken

**Affiliations:** 1 Nutritional Epidemiology Group International Agency for Research on Cancer World Health Organization Lyon France; 2 Department of Behavioural Science and Health University College London London United Kingdom; 3 Leeds Institute of Health Sciences University of Leeds Leeds United Kingdom

**Keywords:** weight loss, habits, mobile apps, mHealth

## Abstract

**Background:**

The Ten Top Tips (10TT) is an intervention based on the habit formation theory that promotes a set of weight management behaviors alongside advice about repetition in a consistent context. Overall, 3 studies have demonstrated that the 10TT can support individuals to lose weight when delivered in a leaflet format. Delivery of 10TT via new technology such as a mobile app could potentially improve its effectiveness and make it more convenient, appealing, and wide reaching.

**Objective:**

This study aimed to provide preliminary indications of the usage, effectiveness, and acceptability of an Android app of the 10TT intervention (Top Tips only app) and a second version including self-regulatory strategies for dealing with tempting foods (Top Tips plus app).

**Methods:**

The 3-month pilot randomized adults with overweight or obesity to (1) Top Tips only app, (2) Top Tips plus app, or (3) waiting list condition. Automated data from app users were collected. Validated questionnaires assessed self-regulatory skills, weight loss (kg), and behaviors at baseline and 3 months. Users’ feedback on their experience using the app was assessed using open questions.

**Results:**

A total of 81 participants took part in the pilot; 28 participants were randomized to the Top Tips only app, 27 to the Top Tips plus app, and 26 to the waiting list condition. On average, participants viewed a mean of 43.4 (SD 66.9) screens during a mean of 24.5 (SD 44.07) log-ins and used the app for 124.2 (SD 240.2) min over the 3-month period. Participants randomized to the Top Tips only app reported the greatest improvement in self-regulatory skills (mean 0.59, SD 1.0), weight loss (mean 4.5 kg, SD 5.2), and adherence to the target behaviors (mean 0.59, SD 0.49) compared with the Top Tips plus (mean_self-regulation_ 0.15, SD 0.42; mean_weight_ −1.9, SD 3.9; and mean_behaviors_ 0.29, SD 0.29) and waiting list condition (mean_self-regulation_ −0.02, SD 0.29; mean_weight_ −0.01, SD 0.51; and mean_behaviors_ 0.08, SD 0.38). Participants who reported the largest improvements, on average, viewed pages 2 to 3 times more, had 2 to 3 times more log-ins, logged their weight 2 to 3 times more, and achieved the tips more than those who reported smaller changes in these outcomes. According to users’ feedback, engagement with the app could be increased by making the app more interactive and allowing more tailoring.

**Conclusions:**

This study suggests that the Top Tips app could potentially be a useful intervention for promoting eating self-regulatory skills, weight loss, and weight management behaviors among adults with overweight or obesity. Future research should develop the app further based on user feedback and test it in larger sample sizes.

**Trial Registration:**

ISRCTN Registry ISRCTN10470937; http://www.isrctn.com/ISRCTN10470937 (Archived by Webcite at http://www.webcitation.org/76j6rQibI)

## Introduction

### Background

Interest is growing in lifestyle interventions that utilize a habit-based approach as they have the potential to promote lasting weight loss and healthy dietary behaviors and are easily scalable. Habit-based interventions promote the repetition of target behaviors in a consistent context to make the behaviors more automatic and habitual [1-3]. These interventions may also promote self-regulatory skills (eg, goal setting, planning, self-monitoring, and feedback on performance) to translate the intended behavior into action and override unwanted automated responses [1,4].

Although weight loss interventions based on habit theory are still scarce [5,6], recent studies using this approach have shown promising results. A total of 3 studies have explored the delivery of a paper-based weight loss intervention that encourages habit formation for a set of target health behaviors, called Ten Top Tips (10TT). In all 3 studies, adults affected by overweight or obesity who received the 10TT leaflet lost significantly more weight (between 1.7 and 3.3 kg) than those allocated to a control group [5,7,8]. A recent study also found that the weight loss promoted by the 10TT was mediated by improvements in both self-regulatory skills and automaticity of the target behaviors [9]. However, the paper format of the 10TT is becoming outdated, and the use of new technology such as mobile apps could potentially encourage engagement among a wider range of users and improve the effectiveness of this intervention.

According to 2 meta-analyses, mobile app interventions can lead to significantly greater weight loss compared with other interventions such as paper-based interventions and counseling and education lesson–based interventions [10-12]. The retention rates of weight loss interventions may also be greater when they are technology-based compared with paper-based format [2]. There are also some indications that the use of new technologies may help people to form healthy habits and break unhealthy ones. Brief technology-based interventions promoting self-regulatory practice have been effective at improving this capacity [13-17]. This, in turn, may help people to form habits. However, most technology-based weight loss interventions currently do not support habit formation [18], and those available are not typically based on theory or evidence [19,20].

Delivering the 10TT weight loss intervention via a mobile app has the potential to be novel, effective, convenient, appealing, cost-effective, and wide reaching. Developing a mobile app version of the 10TT also offers an opportunity for testing out additional components, which could enhance the effectiveness of the intervention. Evidence suggests that strategies such as engaging in pleasant imagery tasks [21], developing intention implementations [22,23], and attention bias [24] could potentially help people to deal with tempting food and, therefore, break existing unhealthy habits. There is also some evidence to support the use of mobile phone apps to break habits through developing implementation intentions and to reduce cravings for unhealthy food through the use of imagery tasks [25]. The addition of a self-regulatory training element to help people deal with food cravings could reduce unhealthy food intake, in addition to the established effects of the 10TT on increasing healthy food intake.

### Objectives

Therefore, this study developed an Android app of the 10TT intervention (Top Tips only app) and a second version that included self-regulatory strategies for dealing with tempting foods (Top Tips plus app). The aim was to provide preliminary indications of the usage, effectiveness, and acceptability of the 2 apps.

## Methods

### Initial Development of the Top Tips App

The development of the Top Tips apps was completed through an iterative process over a period of 1 year, involving 3 main phases: (1) initial development, (2) user testing, and (3) pilot testing.

Both the content and format of the Top Tips apps were developed based on (1) the 10TT leaflet [7], (2) the principles of habit theory, (3) empirical evidence from the field of weight loss and behavioral nutrition, and (4) the experience of the developers in designing health apps for behavior change. They were designed for Android devices as users of these devices tend to have greater socioeconomic variability compared with iPhone operation system users [26]. The team of researchers and app developers met regularly during the development process and agreed to keep the Top Tips apps simple, including only the essential features (see Multimedia Appendix 1), to allow a flexible development process and also because of budget constraints. Although the branding was kept in line with the 10TT leaflet, some necessary changes were made to develop a coherent, well-structured, and attractive app that maximized engagement with the target population.

To encourage habit formation, the apps advised users to make context-specific plans to turn each tip into a habit and adjust these whenever needed. Example plans for each tip were provided. The app also asked users to track their weight in kg and adherence to the tips each day. The apps provided automatic updates of how many times each tip was achieved per week as well as daily reminders to promote engagement with the app. A total of 9 notifications were designed related to different functions of the app, for example, “Don’t worry if you forgot to log anything last week, it’s easy to add to past days—why don’t you start now?”. A random notification was sent each day in the evenings as it was anticipated that this would be the most likely time people would log their adherence to the target behaviors and review their plans. However, participants could turn this function off if they wanted.

The Top Tips plus app included an additional tip targeting self-regulatory strategies to resist tempting food. This new tip was developed based on the current evidence for reducing unhealthy food cravings and avoiding lapses [22-24]. The tip promoted visual imagery and distraction strategies to avoid cues that elicit urges to eat unhealthy foods, which may increase the likelihood of resisting tempting food. The additional tip also provided examples of forming coping plans using these strategies. In line with the other tips, users were required to make their own coping plans to resist unhealthy food and monitor their progress every day, assessing whether they experienced food cravings and whether they could resist them (Multimedia Appendix 2).

### User Testing

The Top Tips only app and the Top Tips plus app were tested with a small convenience sample of adults who owned an Android phone. The user testing aimed to assess preliminary functionality and usability of the Top Tips apps. A total of 8 (63%[5/8] female) people took part in this study, of which 4 tested the Top Tips only app and 4 tested the Top Tips plus app. Participants were invited to download the latest version of the app and were given an individual passcode. They were instructed to enter at least one plan, log completed tips and their weight, check the content of the app for spelling errors, and provide feedback on their experience of the apps and any technical flaws.

Overall, participants reported that they liked the app and found it neat, user-friendly, and attractive. Although the app worked well for most participants, the following issues were raised: (1) inability to enter decimals to their weight in kg, (2) technical difficulties for 2 specific types of Android phones, and (3) difficulty in understanding how to use the app because there was no tutorial. These technical and weight recording issues were fixed in the final versions of the apps. To assist participants with downloading and navigating the app, a PDF document with instructions and a tutorial lasting less than 3 min explaining how to use the app were developed for each version (Multimedia Appendices 3 and 4).

**Figure 1:**
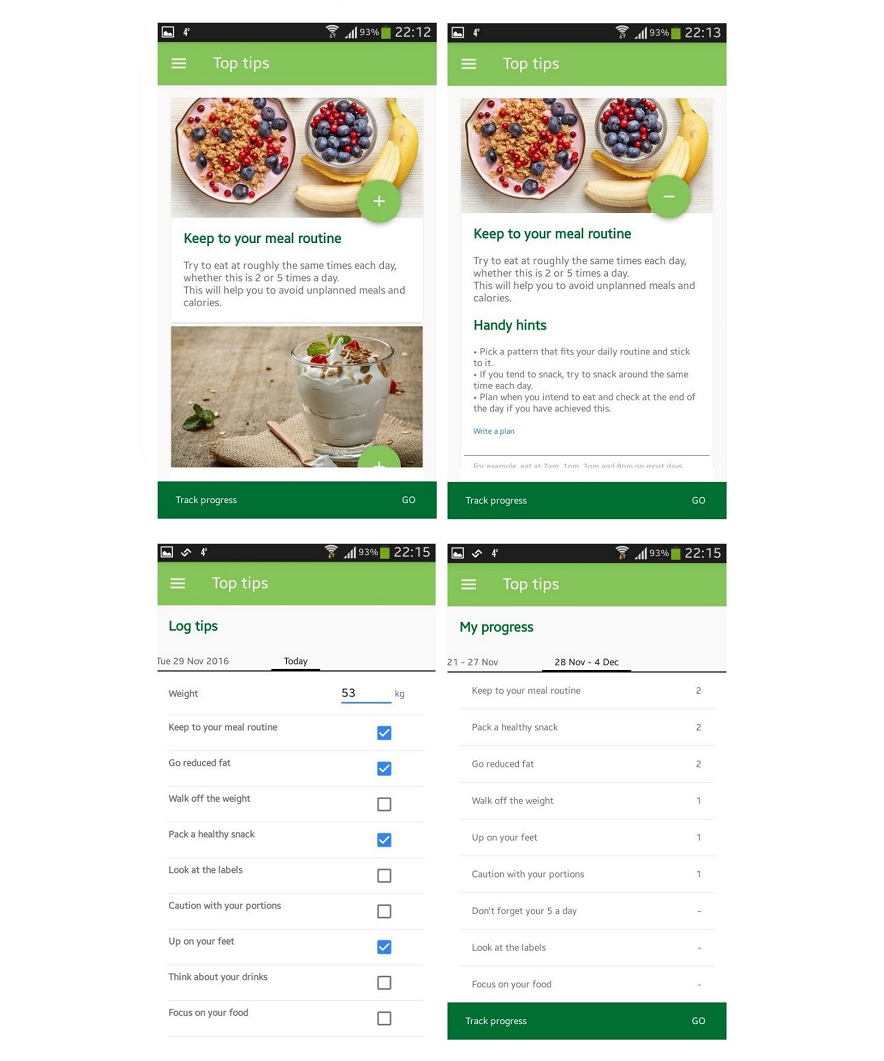
Screenshots of the Top Tips only app.

**Figure 2:**
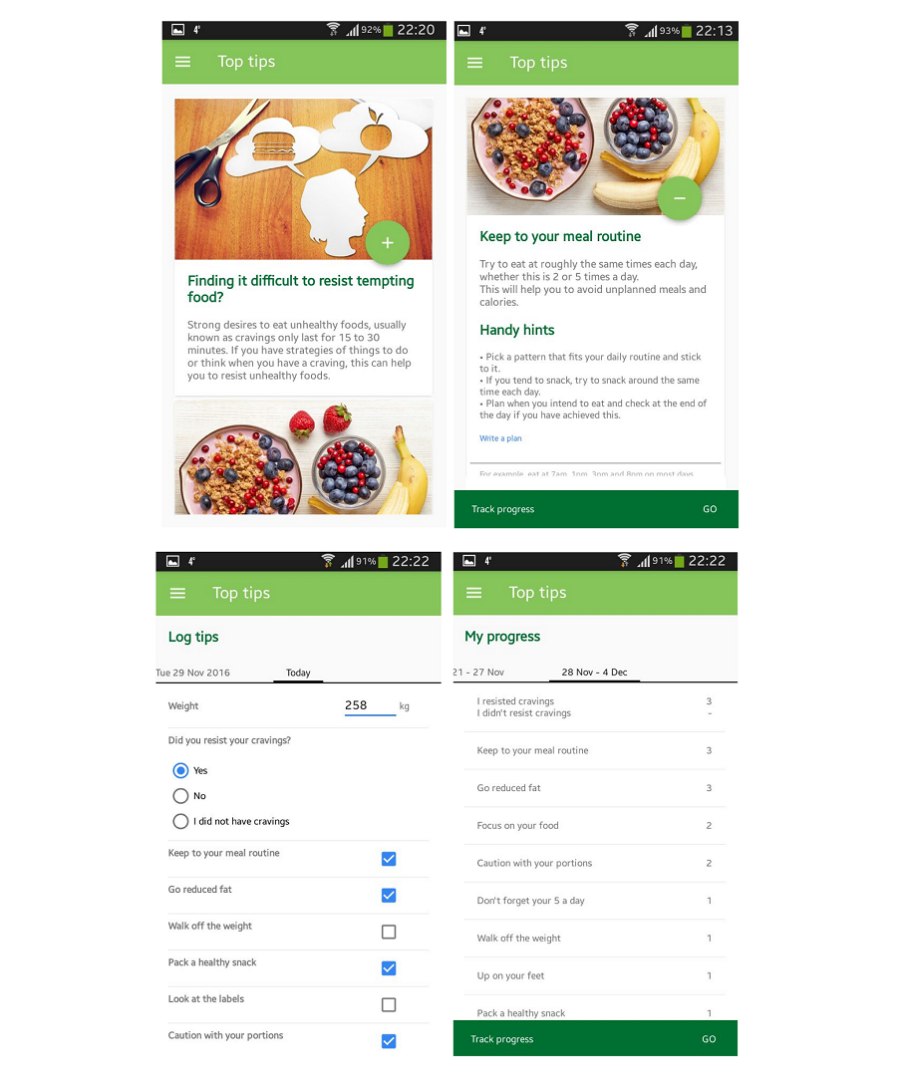
Screenshots of the Top Tips plus app.

The final versions of the 2 Top Tips apps were released on the Google store for pilot testing. Screenshots of the tips, planning, daily tracking, and automatic feedback features for the Top Tips only app are shown in Figure 1, whereas Figure 2 shows these features for the Top Tips plus app.

### Pilot Testing

The pilot was a 3-arm, individually randomized, controlled study in an online sample of adults with overweight and obesity comparing (1) Top Tips only app, (2) Top Tips plus app (including an additional tip on dealing with tempting foods), and (3) no intervention control group (waiting list). The active intervention period was 3 months, and follow-up data were collected at the end of this period. The study was approved by the University College London Ethics Committee (study ID: 5766/003). It also followed the Consort extension for pilot studies (Multimedia Appendix 5)

#### Participants and Recruitment

Participants were eligible to take part in the study if they (1) were adults (18 years or older) from the United Kingdom, (2) owned an Android mobile phone (3) could read English fluently, and (4) were overweight or obese (body mass index [BMI] ≥25 kg/m^2^). Participants were excluded if they (1) were unable to provide informed consent, (2) were pregnant or breastfeeding, (3) were expecting to undergo bariatric surgery in the following 3 months or were recovering from a bariatric surgery, or (4) were on a strict weight loss treatment, such as meal replacements. No upper age limit was established in line with the 10TT leaflet trial [7].

Potential participants were invited via recruitment posters, social media, recruitment websites, and snowball sampling via personal contacts. A research website was also set up to provide interested participants with additional information about the study. Interested participants were invited to fill out an online survey where they were screened for eligibility. Recruitment took place over 2 months, from the beginning of January to the beginning of March 2017.

Eligible participants who gave informed consent and completed the baseline questionnaire were individually randomized to 1 of the 3 group conditions: (1) Top Tips only app, (2) Top Tips plus app, and (3) waiting list. Randomization was performed using the Minimpy software (Sourceforge) [27] and was stratified by gender, age, and BMI classification.

#### Sample Size

A rule of thumb for the sample size of pilot studies is to have around 25 participants per randomization group for a small standardized effect size of 0.2 [28]. As this study involved 3 experimental groups, we aimed to recruit at least 75 participants.

#### Procedure

After randomization, participants randomized to 1 of the 2 app groups received an email with instructions about the intervention and a passcode to access the app. Participants were instructed to use the app every day for 3 months, which is the period usually required to form habits [1,2]. Participants randomized to the control condition received an email explaining that they had been allocated to the waiting list group and that they would receive access to the weight loss app in 3 months’ time. Before randomization and at 3-month follow-up, all participants were requested to complete an online questionnaire. To promote completion of the postintervention assessment, all participants had the chance to enter a draw to win 1 of 3 £20 High Street vouchers. However, they were only informed about the prize draw at the end of the intervention, to ensure that only participants who were motivated to lose weight and to improve their diet were recruited to the study. Participants from the Top Tips apps conditions were also invited to answer qualitative questions at the end of the online follow-up questionnaire to further explore their experience of using the apps.

#### Measures

Automated data from Top Tips only and Top Tips plus users were collected over the 3-month intervention period to assess usage patterns. This included data on the number of log-ins, pages viewed, plans made, and the total time spent on the apps in minutes. Information was also collected on the number of times that weight was logged and each tip was achieved. Information was collected from the Top Tips plus users on the number of times they resisted food cravings, number of times they did not resist, and number of times they did not have food cravings.

The online questionnaires at baseline and follow-up collected information on sociodemographics, self-regulation, anthropometrics, and dietary behaviors. Participants were asked to report their gender, age, ethnicity, marital status, education, and employment status. Due to the small sample size, all variables were categorized into 2 groups. Ethnicity was categorized as *white* or *other* (black, Asian, mixed, or other). Marital status was categorized as *married* (or living as married) and *not married or other* (single, separated, divorced, or widowed). Education was categorized as *nondegree* or *degree*. Employment status was categorized as *paid work* and *unpaid work or other* (unemployed, homemaker, voluntary work, disabled or too ill to work, student, or retired).

Eating self-regulatory skills were assessed using the 5-item Self-Regulation of Eating Behavior Questionnaire [29]. Total mean score and changes over 3 months were calculated. Weight and height were self-reported. For those who did not complete the follow-up questionnaire, their last weight logged on the app was used. Changes in weight in kg over 3 months were calculated. BMI was also calculated and then categorized into overweight (25-29.9 kg/m^2^) or obese (30 kg/m^2^ or over).

Frequency questions were based on those used within a previous 10TT randomized controlled trial (RCT) [7] and assessed the 10 eating and activity behaviors plus self-weighing targeted in the Top Tips apps. For some of these behaviors, more than 1 frequency question was used to better assess adherence to the behavior. For example, for the *look at labels* behavior, 2 questions were generated to ask about how often people looked at labels when (1) preparing food and (2) buying food. A total of 16 questions were used to assess the frequency of carrying out each of the target behaviors over the previous 2 weeks on a 5-point Likert scale. The overall mean score for the 16 behaviors was calculated as well as the mean change from baseline to 3-month follow-up. Dietary intake was assessed in more detail using validated food frequency questionnaires. For example, fat intake was assessed using the dietary fat scale from the validated Dietary Instrument for Nutrition Education [30], which was adapted to broaden the range of ethnically diverse foods and the main components of the UK diet. Fruit and vegetable intake was assessed using 2 validated food frequency questions [31], measuring intake on a 7-point response scale. Similarly, 2 food frequency questions assessed sweet snack (SS) intake, including foods such as chocolates, sweets biscuits, cakes, buns, pastries, and ice-cream. In addition, 4 frequency questions assessed the consumption of sugary sweetened drinks (SSD) intake, including nondiet fizzy drinks, sugar-containing squashes, milkshakes, and hot chocolate. The response options ranged from 1 (never or rarely) to 7 (3 or more times a day). Following the study by McGowan et al (2012), answers were recoded to represent daily intake, for example, 2 to 3 times a week was coded as 0.36. The mean scores for fruit and vegetable, SS, and SSD frequencies were calculated as well as the mean change from baseline to 3-month follow-up.

To assess acceptability of the Top Tips apps, 8 open questions relating to users’ experience of using the apps were included in the online follow-up questionnaire but stated as optional (Multimedia Appendix 6). This included their overall views toward the app, if there was anything that they disliked or found hard to use, if there was anything they liked or found easy to use, if there was anything that they were expecting to see but did not, how the app could be improved, and if they had any other comments they would like to make.

#### Statistical Analyses

For this pilot study, usage pattern and users’ feedback were the primary variables of interest, but the impact of the intervention on self-regulatory skills, weight loss, and behaviors was also explored. All analyses were conducted on an intention-to-treat basis, with participants analyzed based on assigned randomization group [32]. Descriptive analyses were used to characterize the sample by study arm. Baseline differences between those who downloaded and did not download the app were tested using Chi-square tests for categorical variables and *t* tests or Mann-Whitney tests for continuous variables. Descriptive analyses were used to show the usage pattern of the Top Tips apps. Mean, SD, median, and total range were reported for each usage metric.

Exploratory descriptive analyses were performed to obtain an early indication of the effect of the Top Tips apps on eating self-regulatory skills, weight, and behaviors, including dietary intake. Initially, a completer analysis was performed using the complete data at baseline and follow-up for each outcome. Participants with more than 20% of missing data at baseline for the self-regulation and target behaviors questionnaires and with any missing data for dietary intake questions were excluded from the analyses. When there were up to 20% missing data for the self-regulation and target behaviors questionnaires, the individual median score was imputed. Within-group changes from baseline to 3 months were described for each outcome, including 95% CI, and the Cohen effect size was calculated. Sensitivity analysis using the last observation carried forward approach was performed to investigate the potential effect of missing responses on effect sizes. Analysis of variance was also conducted using the imputed data. Descriptive analyses were used to assess the relationships between overall app usage and changes in eating self-regulatory skills, weight, and target behaviors over 3 months. For this analysis, the levels of change in self-regulatory skills, weight, and target behaviors were categorized into 2 groups using ranked percentiles: (1) percentile <75 represented medium-to-small changes and (2) percentile ≥75 represented large changes. Rank percentiles were used to categorize data into low and high as the data were skewed.

Users’ feedback on their experience using the app was analyzed using thematic analysis [33]. This method identifies and reports patterns (themes) within data. All quantitative analyses were undertaken using SPSS version 24.0 (SPSS Inc).

## Results

### Participants and Recruitment

A total of 201 adults were interested in the intervention and were assessed for eligibility. Of these, 120 were excluded because they had a BMI <25.0 kg/m^2^ (n=10), did not own an Android mobile phone (n=81), or did not complete the baseline questionnaire (n=29). A total of 81 participants were eligible to take part in the study; 28 participants were randomized to the Top Tips only app, 27 to the Top Tips plus app, and 26 to the waiting list group. Multimedia Appendix 7 displays the flow diagram of study participation over the 3-month study period.

Table 1 shows the baseline characteristics of the participants, which appeared similar across the 3 study arms. The majority of the participants were female (approximately 90%) and white (approximately 84%). Approximately two-thirds had a degree (approximately 74%) and half were married (approximately 54%) and were in paid work (approximately 59%). Overall mean age was 42.4 (SD 13.4) and BMI was 34.3 kg/m^2^ (SD 7.0). The Top Tips app was downloaded by 60% (17/28) of the participants randomized to the Top Tips only condition and by 70% (19/27) of those randomized to the Top Tips plus condition. Those who did not download the app were not significantly different at baseline with regard to any of the sociodemographic variables from those who downloaded the app.

**Table 1:** Baseline characteristics of the condition groups.

Characteristics		Top Tips only (n=28)	Top Tips plus (n=27)	Waiting list (n=26)
**Gender, n (%)**				
	Female	24 (86)	25 (93)	24 (92)
Age (years), mean (SD)		43.6 (13)	44.0 (14)	40.6 (13)
**Ethnic group, n (%)**				
	White	23 (82)	23 (85)	22 (85)
	Other^a^	5 (18)	4 (15)	4 (15)
**Marital status, n (%)**				
	Married^b^	15 (54)	15 (55)	14 (54)
	Not married or other^c^	13 (46)	12 (44)	12 (46)
**Education, n (%)**				
	Nondegree^d^	6 (21)	8 (30)	7 (27)
	Degree^e^	22 (79)	19 (70)	18 (69)
	Missing^f^	—	—	3.8 (1)
**Employment situation, n (%)**				
	Paid work^g^	20 (71)	15 (56)	13 (50)
	Unpaid work or other^h^	8 (29)	12 (44)	12 (46)
	Missing^f^	—	—	1 (4)
**Weight status, n (%)**				
	Overweight^i^	8 (29)	7 (26)	7 (27)
	Obese^j^	20 (71)	20 (74)	19 (73)
Body mass index, mean (SD)		33.7 (7)	35.0 (8)	34.0 (7)
Eating self-regulatory skills^k^, mean (SD)		2.81 (0.57)	2.87 (0.69)	2.85 (0.51)

^a^Black, Asian, mixed, or other.

^b^Married or living as married.

^c^Single, separated, divorced, or widowed.

^d^Primary/secondary school or O level/GCSEs/A levels or technical/trade certificate/diploma.

^e^Degree or postgraduate degree.

^f^No response.

^g^Employed full-time/employed part-time/self-employed.

^h^Unemployed/full-time homemaker/unpaid or voluntary work/disabled or too ill to work/student/retired.

^i^BMI from 25.0 kg/m^2^ to 29.9 kg/m^2^.

^j^BMI 30.0 kg/m^2^ or over.

^k^Eating self-regulatory skills assessed using the Self-Regulation of Eating Behavior Questionnaires.

### Usage Pattern

Usage pattern for each Top Tip app and overall is presented in Table 2. Although there was significant variability between participants, on average, participants viewed a mean of 43.4 (SD 66.9) screens during a mean of 24.5 (SD 44.07) log-ins and used the app for 124.2 (SD 240.2) min over the 3-month intervention. Plans were made on average 4.6 (SD 3.9) times, weight was logged around 8.3 (SD 15.9) times, and tips were achieved on average 10.1 (SD 21.2) times over the course of the intervention. Participants randomized to the Top Tips only condition seemed to have used the app twice as much as those randomized to the Top Tips plus condition.

**Table 2:** Usage pattern per app and overall.

Usage pattern		Top Tips only (n=17)	Top Tips plus (n=19)	Overall (n=36)	
		Mean (SD)	Mean (SD)	Mean (SD)	Minimum-Maximum observations
Number of screens viewed		56.6 (94.9)	32.2 (24.9)	43.4 (66.9)	2-283
Number of log-ins		33.8 (63.1)	16.5 (13.5)	24.5 (44.07)	1-253
Cumulative minutes using the app		162.1 (296.5)	92.1 (181.4)	124.2 (240.2)	0.01-1200.8
Number of plans made		4.8 (3.9)	4.4 (3.9)	4.65 (3.9)	0-11
Number of times weight was logged		9.9 (20.8)	6.8 (10.3)	8.3 (15.9)	0-74
Number of times tips were achieved		14.0 (29.0)	6.7 (9.9)	10.1 (21.2)	0-102
**Number of times each tip was achieved**					
	Keep to your meal routine	0.06 (0.24)	0.35 (0.81)	0.22 (0.63)	0-3
	Go reduced fat	0.18 (0.52)	0.25 (0.55)	0.22 (0.53)	0-2
	Walk off the weight	0.00 (0.00)	0.00 (0.00)	0.00 (0.00)	0-0
	Pack a healthy snack	0.12 (0.48)	0.15 (0.36)	0.14 (0.42)	0-2
	Look at the labels	0.18 (0.53)	0.25 (0.71)	0.22 (0.63)	0-3
	Caution with your portions	12.4 (28.4)	4.4 (6.9)	8.1 (20.0)	0-100
	Up on your feet	0.06 (0.24)	0.10 (0.31)	0.08 (0.27)	0-1
	Think about your drinks	0.06 (0.24)	0.10 (0.31)	0.08 (0.27)	0-1
	Focus on your food	0.29 (0.47)	0.35 (0.59)	0.32 (0.53)	0-2
	Don’t forget your 5-a-day	0.65 (1.0)	0.85 (1.75)	0.76 (1.46)	0-7
	Extra: Cravings were resisted^a^	—^b^	4.6 (6.8)	4.6 (6.8)	0-25
	Extra: Cravings were not resisted^c^	—^b^	3.2 (4.6)	3.2 (4.6)	0-16

^a^Number of times people resisted their food cravings.

^b^No data for the Top Tips only app as it did not have these extra tips.

^c^Number of times participants did not resist their food cravings.

The tip most frequently achieved was *Caution with portions* (mean 8.1, SD 20.0), followed by *don’t forget your 5 a day* (mean 0.76, SD 1.46) and *Focus on your food* (mean 0.32, SD 0.53). The tip least achieved was *Walk off the weight,* which was not achieved by any participant during the entire intervention. This pattern was found in both apps. Regarding the tip on how to resist tempting food within the Top Tips plus app, participants logged success (mean 4.6, SD 6.8) more times than failure (mean 3.2, SD 4.6) for their attempts to resist tempting food.

### Postintervention Effect on Eating Self-Regulatory Skills, Weight, and Behaviors

**Figure 3:**
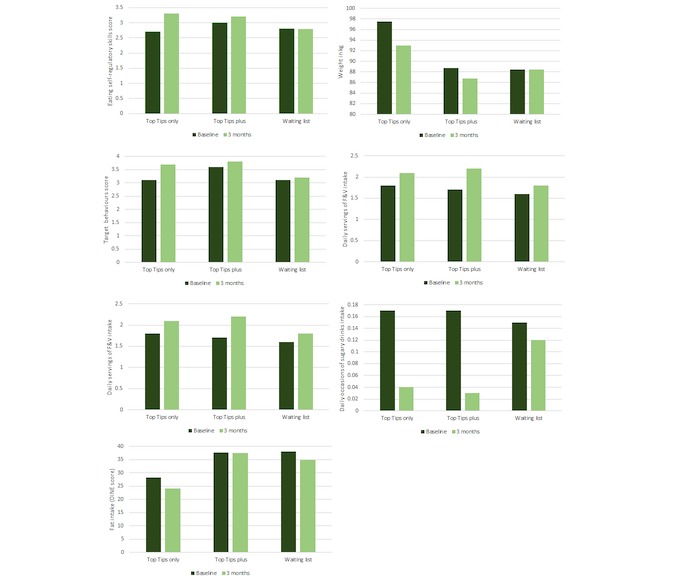
Baseline and follow-up weight and behaviors per condition group. F&V: Fruit and Vegetables.

Baseline and follow-up data for each outcome per group are illustrated in Figure 3, whereas changes over time are shown in Table 3. Eating self-regulatory skills increased the most in the Top Tips only group (mean 0.59, SD 1.0), followed by the Top Tips plus group (mean 0.15, SD 0.42). No changes were found for the waiting list group (mean −0.02, SD 0.29). These changes represented a medium-sized effect for the Top Tips only and small-sized effect for the Top Tips plus condition, which were in line with the effect sizes found in the sensitivity analysis (Multimedia Appendix 8).

**Table 3:** Preliminary indication of the effect of the Top Tips apps on weight loss and behaviors.

Changes over 3 months	Top Tips only (n=17)			Top Tips plus (n=19)			Waiting list (n=26)		
	n (%)	Mean (95% CI)	*d* ^a^	n (%)	Mean (95% CI)	*d* ^a^	n (%)	Mean (95% CI)	*d* ^a^
Self-regulation^b^	5	0.59 (−0.76 to 1.94)	0.53	8	0.15 (−0.20 to 0.50)	0.35	18	−0.02 (−0.16 to 0.12)	0.06
Weight in kg	7	−4.50 (−0.93 to 0.27)	0.80	11	−1.90 (−4.4 to 0.43)	0.05	8	−0.15 (−5.24 to 4.95)	0.002
Target behaviors^c^	6	0.59 (0.06 to 1.11)	1.10	8	0.29 (0.05 to 0.53)	1.00	20	0.08 (−0.09 to 0.26)	0.22
Fruit and vegetable intake^d^	5	0.26 (−0.65 to 1.18)	0.35	8	0.42 (−0.39 to 1.23)	0.43	18	0.22 (−0.29 to 0.73)	0.21
Sweet snacks intake^e^	5	−0.04 (−0.17 to 0.08)	0.45	8	−0.29 (−0.84 to 0.26)	0.43	18	−0.18 (−0.44 to 0.07)	0.35
Sugary sweetened drinks intake^f^	5	−0.07 (−0.17 to 0.04)	0.78	8	−0.13 (−0.41 to 0.14)	0.40	18	−0.03 (−0.08 to 0.02)	0.25
Fat intake^g^	5	−4.20 (−14.1 to 5.74)	0.52	8	−0.12 (−10.84 to 10.59)	0.01	18	−3.10 (−7.20 to 0.87)	0.04

^a^Cohen *d* effect size.

^b^Eating self-regulatory skills assessed using the Self-Regulation of Eating Behavior Questionnaire, scores ranged from 1 (never) to 5 (always).

^c^Overall mean score for the frequency of the 16 target behaviors, scores ranged from 1 (none of the time) to 5 (all of the time).

^d^Fruit and vegetable intake in servings per day.

^e^Daily occasions of sweet snacks intake.

^f^Daily occasions of sugary sweetened drinks intake.

^g^Score for the Dietary Instrument for Nutrition Education questionnaire. Cutoffs: <30 low fat, 30-40 medium fat, and >40 high fat.

The results also suggest that over the 3-month period, weight loss was greater among those who received the Top Tips only (mean −4.5 kg, SD 5.2), followed by those who received the Top Tips plus (mean −1.9 kg, SD 3.9), and no weight loss was observed among those allocated to the waiting list (mean −0.01 kg, SD 0.51). This represented a large-sized effect for the Top Tips only and a medium-sized effect for the Top Tips plus, but according to the sensitivity analysis, the effect on weight loss was small for both app conditions (Multimedia Appendix 8).

Similarly, the Top Tips only app appeared to promote a greater increase in adherence to the target behaviors (mean 0.59, SD 0.49) than the Top Tips plus app (mean 0.29, SD 0.29), whereas no changes were observed in the waiting list (mean 0.08, SD 0.38) condition. These changes represented a large-sized effect for both app conditions. However, the sensitivity analysis suggested that the effect of both apps on adherence to the target behaviors represented a medium-sized effect (Multimedia Appendix 8).

Regarding the effect on dietary intake, the Top Tips plus group experienced the greatest increase in fruit and vegetable intake (mean 0.42, SD 0.97) and decrease in SS (mean −0.29, SD 0.66) and SSD (mean −0.13, SD 0.33) intake (all representing a medium-sized effect) compared with Top Tips only and waiting list groups. With respect to fat intake, the Top Tips only group reported the greatest changes (mean −0.42, SD 8.01, representing a medium-sized effect) compared with the Top tips plus and waiting list groups. Sensitivity analyses suggested a small effect size for all dietary changes (Multimedia Appendix 8).

None of the changes in the outcomes between the condition groups were found to be significant in the sensitivity analysis (Multimedia Appendix 8). However, this should be interpreted with caution as this study was not powered to detect significant differences.

### Relationships Between App Usage and Changes in Eating Self-Regulatory Skills, Weight, and Behaviors

Table 4 shows the relationships between the Top Tips apps usage and changes in self-regulatory skills, weight, and adherence to target behaviors. The results suggest that participants with the greatest changes for these outcomes, on average, viewed pages 2 to 3 times more, had 2 to 3 times more log-ins, logged their weight 2 to 3 times more, and achieved the tips more than those who showed smaller changes in these outcomes. Moreover, participants who reported the greatest changes in eating self-regulatory skills, weight, and adherence to target behaviors made on average 1, 2, and 3 plans less than those with smaller changes, respectively. App usage in minutes was also higher among those with greater improvements for eating self-regulatory skills (approximately 500% higher) and target behaviors (approximately 140% higher) than those who made smaller changes. In contrast, those who lost more weight used the apps about 15% less than those who lost less weight over the course of the intervention.

**Table 4:** App usage per level of changes in self-regulatory skills, weight, and target behaviors over 3 months (data from both Top Tips apps).

Outcome/app feature		Percentile <75^a^, changes over 3 months			Percentile ≥75^a^, changes over 3 months		
		n	Mean (SD)	Median	n	Mean (SD)	Median
**Self-regulation^b^**							
	All participants^c^	8	−0.06 (0.58)	−0.10	5	0.92 (0.58)	0.80
	Number of screens viewed	8	41 (26)	37	5	84 (113)	41
	Number of log-ins	8	22 (14)	18	5	38 (45)	22
	Cumulative minutes using the app	8	48.6 (57.8)	15.9	5	241.6 (339.1)	64.6
	Number of plans made	8	6 (4)	7	5	5 (5)	8
	Times weight was logged	8	7 (4)	6	5	24 (32)	6
	Times tips were achieved	8	7.7 (4.3)	5	5	23.2 (30.6)	9
**Weight^d^**							
	All participants^c^	6	1.13 (2.09)	0.97	12	−4.97 (4.05)	−3.65
	Number of screens viewed	6	62 (63)	6	12	85 (101)	12
	Number of log-ins	6	26 (21)	21	12	53 (73)	32
	Cumulative minutes using the app	6	212.9 (315.7)	50.3	12	184.8 (350.6)	60.6
	Number of plans made	6	7 (4)	8	12	5 (4)	6
	Times weight was logged	6	9 (9)	6	12	21 (24)	10
	Times tips were achieved	6	10.3 (11.2)	7	12	25.5 (33.2)	10
**Target behaviors^e^**							
	All participants^c^	7	0.12 (0.26)	0.25	7	.73 (.26)	.62
	Number of screens viewed	7	38 (20)	39	7	109 (122)	72
	Number of log-ins	7	18 (11)	19	7	69 (89)	44
	Cumulative minutes using the app	7	175.8 (310.6)	35.9	7	242.0 (438.3)	64.6
	Number of plans made	7	7 (4)	7	7	4 (5)	2
	Times weight was logged	7	7 (4)	6	7	25 (28)	10
	Times tips were achieved	7	7.5 (5.13)	7	7	32.4 (39.9)	10

^a^Changes to the outcome over 3 months categorized according to the percentile, that is, <75=medium to low changes and ≥75=greater changes.

^b^Changes in eating self-regulatory skills assessed using the Self-Regulation of Eating Behavior Questionnaire

^c^Data from Top Tips only and Top Tips Plus participants.

^d^Changes in weight in kg.

^e^Changes in the overall mean score for the frequency of the 16 target behaviors.

### Acceptability Feedback

A total of 8 participants gave feedback on their experience using the Top Tips apps. Of these, 75% were female (n=7). Two participants complained about technical issues: one participant reported an issue in downloading the app and they were, therefore, unable to follow the intervention, and the other participant was unable to access the daily tips.

Participants’ overall views toward the app were both positive and negative. Some participants mentioned that they did not find the app useful and found it unoriginal and boring. Others said the app was well designed and helped them to track their diet plan:

It is very well designed. It helps you to keep track of your weight loss goals.

Male, 30 years old

I didn't find it particularly helpful.

Female, 43 years old

Helped me focus on my diet plan.

Female, 57 years old

Boring, unoriginal and old hat.

Female, 58 years old

Participants also commented on what they liked and found easy to use. The way the tick boxes were designed to track their adherence to the target behaviors was considered effective and easy to use. Some participants also mentioned that they liked the daily reminders and the possibility of setting their own plans:

[I liked the] daily weight reminder.

Female, 57 years old

The way you have to tick boxes. Easily and effective. It helps you to build new eating habits.

Male, 30 years old

In contrast, some participants said they disliked the reminder, as they found it annoying. The lack of interactivity was also mentioned as a negative aspect of the Top Tips app and the fact that they could not tailor the app to their personal needs:

Lack of any interactivity.

Female, 50 years old

Absence of feedback support.

Female, 58 years old

Couldn't delete the goals I didn't like.

Female, 43 years old

With respect to users’ expectations, some participants said that the app was just not what they expected. Some were expecting the app to include a food diary and allow them to tailor the goals. Some were also expecting that the app would involve more complex information related to weight loss:

Just wasn't what I expected.

Female, 56 years old

New ideas motivating information on metabolism and food and exercise. Your app had the standard I would expect from a gcse student.

Female, 58 years old

Ability to tailor goals more.

Female, 43 years old

Finally, participants made some suggestions for improving the Top Tips app. Some participants suggested the inclusion of recipes and the use of different strategies to remind people about the tips apart from the daily notifications, such as emails. They also suggested the inclusion of food diaries to track their dietary intake:

Reminders should come up at different times—and also try different strategies (notifications, e-mail, etc.). It could [also] include healthy recipes to help people cook healthy food at home.

Male, 30 years old

Something more like my fitness pal.

Female, 50 years old

## Discussion

### Principal Findings

The findings of this study suggested that the Top Tips habit-based app could potentially be a useful intervention for promoting eating self-regulatory skills, weight loss, and healthy behaviors among adults with overweight or obesity. The usage patterns indicated those who engaged more with the app also reported greater changes in self-regulatory skills, weight, and adherence to target behaviors. Although there are hundreds of commercially available mobile phone apps designed to help people lose weight or form habits, most of these are neither theory nor evidence based [19]. Recently, a study assessed the feasibility of the Habit app for weight loss problem solving [34]. However, this study did not include all the self-regulatory components required to form habits, such as self-monitoring, and neither did it assess the effect of the app intervention on automaticity or dietary behaviors. Therefore, this is one of the first studies to provide some indications of the usage and effect of a weight loss app based on the habit formation theory.

The app was expected to be accessed every day over 3 months to log achieved tips and current weight, but it was accessed on average 25 times. This is in agreement with a systematic review that suggested that most mobile app interventions for weight loss interventions tend to have high attrition and participants tend to disengage from the app after the first month [10]. Furthermore, on average, people made plans for half of the tips, and most of the tips were achieved less than 3 times over the course of the intervention. The exception was the tip on *Caution with portions*, which was achieved on average 8 times, and weight, which was also logged 8 times on average. The tip *walk off the weight* was not achieved by anyone. However, this does not mean people did not increase the number of steps because of this intervention, as they might have increased but not reached the 10,000 steps recommended per day. The integration of an electronic activity monitor to the app could help to better understand the effect of the intervention on activity behaviors. Overall, this usage pattern suggests that there is room for improvement regarding engagement with the app.

According to users’ feedback, engagement with the app could be increased by making the app more interactive, enabling more tailoring to personal needs, and including more resources for weight loss (eg, recipes). The app’s simplicity and design should be maintained, as these were aspects considered positive by users. A recent study highlighted these features as important for keeping users engaged, alongside other features such as feedback function, ability to change design to suit own preference, and tailored information [10]. In-depth focus groups with the target population could also help to better understand the aspects necessary to improve the app. In addition, a better understanding of engagement should also be considered by using the recently published conceptual framework of engagement with digital behavior change interventions (DBCIs), which states that engagement may be influenced by the DBCI itself, the context of use, mechanisms of action of the DBCI, and the target behavior [35]. Finally, considering that only 8 participants opted to answer the feedback questions, future studies should consider including closed feedback questions to increase the response rate and improve our understanding of users’ experiences of these apps.

There was significant interest in the study: over 2 months, 201 people signed up for the study. Of these, 81 adults with overweight or obesity were excluded because they did not have an Android phone. Among those randomized to the app conditions, about one-third did not download the app, suggesting a reduction in interest before even beginning the intervention. The responses for the follow-up online questionnaire were also very low for the app conditions (approximately 25%). In contrast, the follow-up response for the waiting list was high (77%), possibly because of the fact that completing the follow-up questionnaire was a condition for subsequent access to the Top Tips app. Future studies should improve the instruction materials and test other strategies to reduce the dropout for the intervention conditions. The inclusion of face-to-face or telephone support before and after technology-based interventions may increase retention and engagement as well as weight loss [11]. In addition, making the app available for iPhone operation system could increase the reach of the intervention and also improve retention [36]. Dual phone-computer access could also help increase retention, as it is valued by users [37]. These were not possible to implement in this study because of budget constraints but could be addressed in future studies.

The majority of participants in this pilot were white, female, and highly educated. This corroborates with findings from weight management interventions, which tend to underrepresent men [38]. Mobile phone ownership also tends to be higher among those more affluent and educated [39,40]. However, the use of mobile phones has been increasing among lower SES populations, reducing social inequalities for access to evidence-based health apps [39]. Future studies should consider recruitment through clinical settings and targeted strategies to try and achieve a more socioeconomically balanced sample.

Although this study was not powered to detect changes or to explore mechanisms of action, the direction of the results observed in this pilot is a preliminary indication that the app worked as expected. For example, participants in both app group conditions improved their eating self-regulatory skills, whereas no changes were observed in the waiting list group. Users more engaged with the self-regulatory components of the app improved their eating self-regulatory skills to a greater extent, had greater adherence to the target behaviors, and lost more weight than those who engaged less. This is in line with recent evidence suggesting that nutrition and weight loss interventions using self-regulation components tend to be more effective [41,42]. Frequency of app use has also been related to higher success in changing diet and activity behaviors [43]. In contrast with these results, the time of using the apps was greater among those who lost less weight. This may reflect the fact that some people left their app open but were not necessarily using it, suggesting that this is a less accurate tool for measuring engagement.

Both the Top Tips only (mean −4.5 kg) and the Top Tips plus (mean −1.9 kg) apps appeared to promote a greater weight loss than the waiting list (mean −0.01 kg). This is in line with findings from a pilot RCT using the 10TT leaflet in a UK community-based sample, which showed that the 10TT group lost 2 kg, whereas the waiting list group lost 0.4 kg over 8 weeks [5]. Similarly, a recent RCT using the 10TT with adults who had overweight or obesity in Australia found a weight loss of 3.3 kg in the 10TT group compared with 0.4 kg in the control group over 12 weeks [8]. In addition, a previous RCT in patients with obesity from UK primary care showed that those who received the 10TT leaflet lost 1.68 kg over 3 months, and this was maintained over 2 years [44]. Regarding the effect on target behaviors and dietary intake, both app groups reported changes in the expected direction, which were in general greater than those reported by the waiting list group. Although the effects of the Top Tips app on weight loss and behaviors are promising, they should be interpreted with caution because of the small sample size. Future studies should also test the longer-term effects of the Top Tips app.

It was not possible to draw conclusions regarding any differences in impact between the app conditions. The Top Tips plus app appeared to promote greater absolute decreases in SS and SSD intake compared with the Top Tips only app, as expected. However, in contrast, the absolute changes in self-regulatory skills, target behaviors, and weight loss appeared greater in the Top Tips only condition. This may reflect differences in usage between the apps, as participants in the Top Tips only group used the app almost twice as much as the Top Tips plus participants. The greater usage patterns among those using the Top Tips only app may simply reflect the small sample size of this study, which increases the chance of false positives and can inflate effect sizes. Therefore, the potential additive effect of this new tip on how to deal with tempting food needs further examination using larger samples sizes. Future studies testing the Top Tips app would also benefit from a variety of experimental designs that tease out the main active components within the intervention, for example, a sequential multiple assignment trial or a multiphase optimization strategy design [45].

### Limitations

This study had a number of limitations. The sample size was small and the study was not powered to detect differences in the outcomes, thus limiting the conclusions that can be drawn from the results. Allocation bias might also have affected the results, as people were not blinded to their condition. Ethnic minorities, men, and people from lower SES backgrounds were underrepresented. There are also limitations related specifically to the measure used to assess dietary intake. The frequency questions lacked portion size information and did not allow the calculation of overall energy intake. This may have limited the accuracy of the data collected and the understanding of changes in dietary intake. However, the unannounced and self-administered features of these questions combined with the fact that they captured habitual behaviors are important strengths of these measures [46]. Furthermore, given that the measures used in this study were all self-reported, changes in self-regulatory skills, weight, adherence, and dietary intake may represent the individuals’ perception of change rather than actual change. Future studies should aim to use real-time mobile-based assessment of nutrition, physical activity, and behaviors, as this may reduce participant burden and bias [47].

### Conclusions

The findings of this paper suggest that an app version of the 10TT habit-based program may potentially enhance self-regulatory skills and promote healthy dietary behaviors and weight loss. Although engagement was moderate, the results indicated that absolute changes in the outcomes increased with app usage, suggesting that it worked better among those who did engage. According to users, the Top Tips app could be improved and engagement encouraged through more interactivity and weight loss resources and by enabling tailoring. Future research should seek to develop the app further and test it in larger, more diverse samples using designs that enable the main active components within the intervention to be examined.

## Appendix

Multimedia Appendix 1 Top Tips app features.

Multimedia Appendix 2 Additional tips on resisting tempting foods.

Multimedia Appendix 3 Tutorial for the Top Tips only app.

Multimedia Appendix 4 Tutorial for the Top Tips plus app.

Multimedia Appendix 5 Consort checklist for pilot studies.

Multimedia Appendix 6 Qualitative questions on users’ experience.

Multimedia Appendix 7 Flow diagram of participation during the 3-month study period.

Multimedia Appendix 8 Sensitivity analysis.

## Abbreviations

10TT: 10 Top Tips
BMI: body mass index
DBCIs: digital behavior change interventions
RCT: randomized controlled trial
SS: sweet and salty snacks
SSD: sugary sweetened drinks
