# Supplementary material for: Development of the Top Tips Habit-Based Weight Loss App and Preliminary Indications of Its Usage, Effectiveness, and Acceptability: Mixed-Methods Pilot Study
Source: JMIR Mhealth Uhealth. 2019 May 10;7(5):e12326. doi: 10.2196/12326 (PMC6533874; doi:10.2196/12326)
Supplement: Multimedia Appendix 1 [file mhealth_v7i5e12326_app1.pdf]

Top Tips app features

Feature	Details
Information about the Top Tips, a shopping guide, and information about habits and being overweight	Allow users to read information about the 10 target behaviours (and an extra tip on dealing with tempting food in the Top Tips ‘plus’ app); handy hints for each tip; shopping guide; being overweight and; how to form habits.
Weekly plan of how to adopt the tips	Allow users to enter their own plans for each tip and edit them at any time as well as seeing examples of plans.
Daily record of which tips have been achieved	Allow users to log their adherence to each tip every day.
Daily record of weight	Allow users to log their current weight in kg every day.
Weekly review of progress	Give automatic feedback on their weekly performance at adhering to the top tips.
Daily notifications reminders	Send daily notifications to increase engagement with the app.
Tracking of individual data	Provide users with individual passcodes in order to track their individual data.
