# Supplementary material for: Development of the Top Tips Habit-Based Weight Loss App and Preliminary Indications of Its Usage, Effectiveness, and Acceptability: Mixed-Methods Pilot Study
Source: JMIR Mhealth Uhealth. 2019 May 10;7(5):e12326. doi: 10.2196/12326 (PMC6533874; doi:10.2196/12326)
Supplement: Multimedia Appendix 2 [file mhealth_v7i5e12326_app2.pdf]

ADDITIONAL ADVICE ON BREAKING HABITS

Finding it difficult to resist tempting food?

Strong desires to eat unhealthy foods, usually known as cravings only last for 15 to 30 minutes. If you have strategies of things to do or think when you have a craving, this can help you to resist unhealthy foods.

You can opt to:

1. Do something else instead

Make plans to distract yourself when you are craving or tempted by unhealthy foods. You can make a plan of doing something else, for example:

- When I'm craving for (food) I'll text a friend of mine or watch a music clip on YouTube.

2. Think about something else instead

You can also make a plan of thinking about your favourite (non-food) activity and focus on that as long as you can, for example:

- If I'm craving for (food), I'll image myself playing piano
