# Supplementary material for: Development of the Top Tips Habit-Based Weight Loss App and Preliminary Indications of Its Usage, Effectiveness, and Acceptability: Mixed-Methods Pilot Study
Source: JMIR Mhealth Uhealth. 2019 May 10;7(5):e12326. doi: 10.2196/12326 (PMC6533874; doi:10.2196/12326)
Supplement: Multimedia Appendix 4 [file mhealth_v7i5e12326_app4.pdf]

Top Tips weight loss app 2 - Tutorial for the Top Tips 'plus' app.

URL: https://youtu.be/n-qd9EZMUMo2017.

Archived at http://www.webcitation.org/75mjFBRnt on January 29th, 2019.
