# Supplementary material for: Development of the Top Tips Habit-Based Weight Loss App and Preliminary Indications of Its Usage, Effectiveness, and Acceptability: Mixed-Methods Pilot Study
Source: JMIR Mhealth Uhealth. 2019 May 10;7(5):e12326. doi: 10.2196/12326 (PMC6533874; doi:10.2196/12326)
Supplement: Multimedia Appendix 6 [file mhealth_v7i5e12326_app6.pdf]

Qualitative questions on users' experience

1. What are your overall views toward the 10TT app?

2. Was there anything you particularly disliked?

3. Was there anything you found particularly hard to use?

4. Was there anything you particularly liked?

5. Was there anything you found particularly easy to use?

6. Anything you wanted to see there/expected to see there but didn't?

7. Do you have any suggestions for how the app could be improved?

8. Are there any other comments you would like to make?
