# Supplementary material for: Development of the Top Tips Habit-Based Weight Loss App and Preliminary Indications of Its Usage, Effectiveness, and Acceptability: Mixed-Methods Pilot Study
Source: JMIR Mhealth Uhealth. 2019 May 10;7(5):e12326. doi: 10.2196/12326 (PMC6533874; doi:10.2196/12326)
Supplement: Multimedia Appendix 7 [file mhealth_v7i5e12326_app7.pdf]

Flow diagram of participation during the 3-month study period

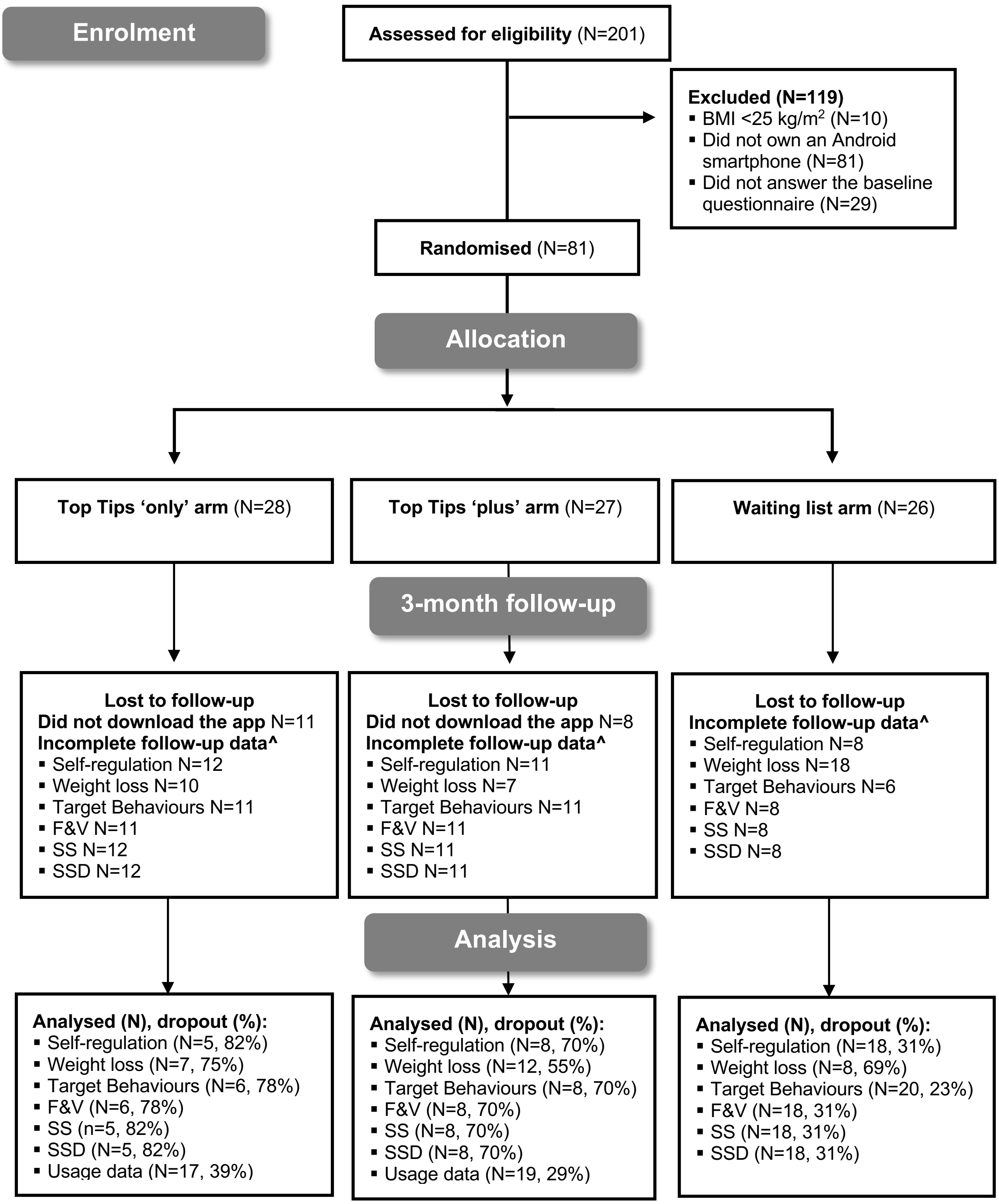


**Note=** Self-regulation refers to data for the Self-Regulation of Eating Behaviour Questionnaire. Target Behaviours refers to data for the frequency of the 16 target behaviours. F&V refers to data for Fruit and vegetable intake in servings per day. SS refers to data for daily occasions of sweet snacks intake. SSD refers to data for daily occasions of sugary sweetened drinks intake. Usage data refers to the automated data obtained from the Top Tips apps. ^Incomplete follow-up data due to lack of recent usage data or incomplete follow-up questionnaire.
