# Supplementary material for: Development of the Top Tips Habit-Based Weight Loss App and Preliminary Indications of Its Usage, Effectiveness, and Acceptability: Mixed-Methods Pilot Study
Source: JMIR Mhealth Uhealth. 2019 May 10;7(5):e12326. doi: 10.2196/12326 (PMC6533874; doi:10.2196/12326)
Supplement: Multimedia Appendix 8 [file mhealth_v7i5e12326_app8.pdf]

Preliminary indication of the effect of the Top Tips apps on weight loss and behaviours: sensitivity analysis

Outcome	Top Tips only					Top Tips plus					Waiting list					
		Baseline	Follow-up	Changes			Baseline	Follow-up	Changes			Baseline	Follow-up	Changes		
	N	M (sd)	M (sd)	M (95%CI)	d¥	N	M (sd)	M (sd)	M (95%CI)	d¥	N	M (sd)	M (sd)	M (95%CI)	d¥	*p*^
Self-regulation[1]	28	2.8(.57)	2.9(.71)	.10(-.08;.29)	.48	27	2.8(.62)	2.9(.62)	.04(-.04;.13)	.19	26	2.8(.51)	2.8(.43)	-.01(-.11;.08)	.04	.439
Weight in kg	28	93.5 (15.4)	92.4(14.6)	-1.1(-2.3;.09)	.35	27	93.1(19.5)	92.3(20.2)	-.78(-1.8;.16)	.29	26	93.6(23.4)	93.6(23.9)	-.004(-1.1;1.1)	.001	.364
Target Behaviours[2]	28	3.0(.35)	3.1(.50)	.12(.-.00;.25)	.38	27	3.2(.51)	3.3(.58)	.09(.00;.16)	.43	26	3.0(.45)	3.1(.48)	.06(-.07;.20)	.19	.746
F&V intake[3]	28	1.77(.87)	1.82(.90)	.04(-.07;.16)	.15	27	1.52(1.1)	1.64(1.1)	.12(-.08;.33)	.22	26	1.50(.71)	1.65(.81)	.15(-.19;.49)	.18	.798
SS intake[4]	28	.63(.73)	.62(.73)	-.01(-.02;.00)	.19	27	.65(.76)	.56(.73)	-.08(-.23;.06)	.23	26	.47(.45)	.34(.21)	-.13(-.30;.04)	.29	.406
SD intake[5]	28	.17(.22)	.16(.22)	-.01(-.02;.00)	.28	27	.16(.26)	.12(.16)	-.04(-.11;.03)	.21	26	.17(.16)	.15(.17)	-.02(-.05;.01)	.21	.209
Fat intake[6]	28	35.4(12.2)	34.7(12.7)	-.75(-2.1;.60)	.21	27	36.8(14.1)	36.7(14.3)	-.03(-2.6;2.6)	.005	26	36.0(11.1)	33.8(10.3)	-2.2(-4.9;.57)	.32	.397

Note=[1]Eating self-regulatory skills assessed using the Self-Regulation of Eating Behaviour Questionnaire, scores ranged from 1 (never) to 5 (always). [2]Overall mean score for the frequency of the 16 target behaviours, scores ranged from 1 (none of the time) to 5 (all of the time). [3]Fruit and vegetable intake in servings per day. [4]Daily occasions of sweet snacks intake. [5]Daily occasions of sugary sweetened drinks intake. [6]Score for the DINE questionnaire - Cut offs: <30 low fat; 30-40 medium fat; >40 high fat. Sensitivity analysis used the Last observation carried forward approach. M= mean. sd=Standard deviation. ¥Cohen's d effect size. ^*p*-value for analysis of variance (ANOVA). 95% Confidence Intervals.
